# Diet-Gene Interactions and PUFA Metabolism: A Potential Contributor to Health Disparities and Human Diseases

**DOI:** 10.3390/nu6051993

**Published:** 2014-05-21

**Authors:** Floyd H. Chilton, Robert C. Murphy, Bryan A. Wilson, Susan Sergeant, Hannah Ainsworth, Michael C. Seeds, Rasika A. Mathias

**Affiliations:** 1 The Center for Botanical Lipids and Inflammatory Disease Prevention, Wake Forest School of Medicine, Winston-Salem, NC 27157, USA; E-Mails: ssergean@wakehealth.edu (S.S.); mseeds@wakehealth.edu (M.C.S.); 2 Department of Physiology/Pharmacology, Wake Forest School of Medicine, Winston-Salem, NC 27157, USA; 3 Molecular Medicine and Translational Sciences, Wake Forest School of Medicine, Winston-Salem, NC 27157, USA; E-Mails: bryawils@wakehealth.edu (B.A.W.); hainswor@wakehealth.edu (H.A.); 4 Department of Pharmacology, University of Colorado Denver, Aurora, CO 80045, USA; E-Mail: Robert.Murphy@ucdenver.edu; 5 Department of Biochemistry, Wake Forest School of Medicine, Winston-Salem, NC 27157, USA; 6 Division of Allergy and Clinical Immunology, Department of Medicine, The Johns Hopkins University, Baltimore, MD 21224, USA; E-Mail: rmathias@jhmi.edu

**Keywords:** polyunsaturated fatty acids, nutrition, genetic variants, fatty acid desaturase (*FADS*), single nucleotide polymorphisms, arachidonic acid, eicosanoids, inflammation, cardiovascular disease

## Abstract

The “modern western” diet (MWD) has increased the onset and progression of chronic human diseases as qualitatively and quantitatively maladaptive dietary components give rise to obesity and destructive gene-diet interactions. There has been a three-fold increase in dietary levels of the omega-6 (*n*-6) 18 carbon (C18), polyunsaturated fatty acid (PUFA) linoleic acid (LA; 18:2*n*-6), with the addition of cooking oils and processed foods to the MWD. Intense debate has emerged regarding the impact of this increase on human health. Recent studies have uncovered population-related genetic variation in the LCPUFA biosynthetic pathway (especially within the fatty acid desaturase gene (*FADS*) cluster) that is associated with levels of circulating and tissue PUFAs and several biomarkers and clinical endpoints of cardiovascular disease (CVD). Importantly, populations of African descent have higher frequencies of variants associated with elevated levels of arachidonic acid (ARA), CVD biomarkers and disease endpoints. Additionally, nutrigenomic interactions between dietary *n*-6 PUFAs and variants in genes that encode for enzymes that mobilize and metabolize ARA to eicosanoids have been identified. These observations raise important questions of whether gene-PUFA interactions are differentially driving the risk of cardiovascular and other diseases in diverse populations, and contributing to health disparities, especially in African American populations.

## 1. Introduction

Perhaps at no time in human history has the human diet changed so dramatically and rapidly than in the past 75 years in developed countries. It is estimated that foods supplying 72% of the dietary calories consumed presently in western diets would not have been found in hunter-gatherer diets [1]. Changes in food type (quality) and quantity found in the modern western diet (MWD) have been largely driven by technological changes in food production and processing to provide high calorie and taste appealing (high sugars, refined grains and oils) foods to large urban populations [1]. Evidence is accumulating that many of these changes have led to detrimental increases in obesity and gene-diet interactions that are responsible for an elevation in localized and systemic inflammation; this inflammation then contributes to a wide range of human diseases including cardiovascular disease, diabetes, cancer, asthma, allergies, chronic joint disease, skin and digestive disorders, dementia and Alzheimer’s disease [2,3,4,5,6,7,8,9]. For example, three decades of research show that high intakes of refined carbohydrates, added sugars and a radical change in the nature of ingested fats, and animal-source foods have dramatically escalated obesity in the developed and now developing world [1]. With regard to fats, animal husbandry has led to production of beef with profoundly abnormal interstitial fat (called “marbling”) and widespread *n*-3 deficiency.

As challenging as obesity and gene-diet interactions have been for overall populations of developed countries such as the US, they manifest themselves in a particularly negative way for certain populations and ethnic groups [10,11,12,13,14,15]. Several lines of evidence now indicate that a disproportionate burden of preventable disease, death, and disability exists in certain racial and ethnic minority populations, especially African Americans. This review examines the current state of our knowledge regarding the relationship between the intake of polyunsaturated fatty acids (PUFAs) from the MWD and the genetics of PUFA biosynthesis and metabolism in distinct human populations. Specifically, this review discusses how the combination of dramatic increases in levels of certain dietary PUFAs together with diet-gene interactions within PUFA pathways may be driving chronic diseases and health disparities.

## 2. Review

### 2.1. A Dramatic Change in the PUFA Content in Our Diet

**Figure 1:**
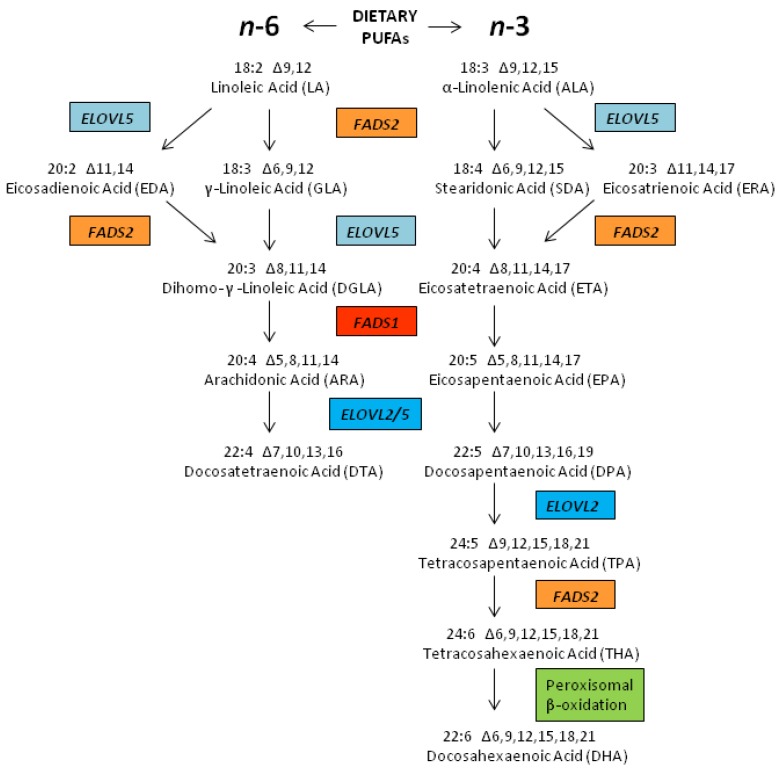
LCPUFA Biosynthetic Pathways. LCPUFA are derived from C18PUFAs (LA and ALA obtained from the mammalian diet) by alternate desaturation (red/orange enzymes) and elongation (blue enzymes) steps. These enzymes utilize both *n*-6 and *n*-3 substrates. *n*-3 LCPUFAs undergo further metabolism through a β-oxidation step (green box) to the generate DHA.

For the purposes of this review, we will discuss the metabolism, genetics and biology of the most abundant *n*-6 or *n*-3 18 carbon (C18) PUFAs and the *n*-6 or *n*-3 long chain (LC; 20–24 carbon) PUFAs. Two C18 PUFAs, linoleic acid (LA, 18:2, *n*-6) and α-linolenic acid (ALA, 18:3, *n*-3) are considered essential fatty acids because they cannot be synthesized by mammals including humans and thus must be obtained from the diet (Figure 1). LA is found in vegetable oil products (soybean, corn, palm, and canola oils as well as margarine and shortenings), and is by far the most abundant PUFA in today’s MWD, contributing more than 90% of ingested PUFAs and 7%–8% of food energy consumed [16]. ALA is found in green plants, nuts and botanical oils, such as flax seed oil, and represents ~1% of food energy.

Following the 1961 American Heart Association Central Committee Advisory Statement to replace saturated fat with PUFAs in the diet, food production companies began replacing the saturated fatty acids in processed foods with unsaturated fatty acid oils, especially soybean oil [17]. As a result, vegetable oils, shortening and margarine were recommended as replacements for animal fats such as butter, cream, and cheese [17]. These changes led to a marked (two to three-fold) increase in dietary LA, an estimated 40% reduction in total *n*-3 LCPUFAs levels, and a large shift in the ratio of dietary *n*-6/*n*-3 C18 PUFAs consumed from ~5:1 to >10:1 [17,18].

### 2.2. A Debate about the Health Impact of n-6 PUFAs

Recently, there has been intense debate in the scientific community over the impact of the recommendation to replace saturated fatty acids with *n*-6 PUFAs and the health effects of raising dietary *n*-6 PUFA levels in general. In favor of recommendations to increase dietary *n*-6 PUFAs, several randomized controlled trials and population cohort studies have shown benefits of *n*-6 PUFAs when measuring cardiovascular disease biomarkers such as serum lipids and lipoproteins [19,20,21]. Based on these studies, the American Heart Association, once again in 2009, recommended human diets should include high levels of *n*-6 PUFAs that comprise at least 5%–10% of the energy intake [22].

On the other side of the argument, Ramsden and colleagues recently re-examined studies utilized to support this recommendation and found that many of the oils used in the aforementioned clinical trials were mixtures of *n*-6 and *n*-3 PUFAs. Their data suggest that only substituting *n*-6 PUFAs for saturated and *trans* fatty acids actually trended toward increased risk of death from all causes [23,24]. This same group also recently reexamined the Sydney Diet Heart Study (458 men aged 30–59 with a recent coronary event), which replaced dietary saturated fatty acids with a high LA-containing diet utilizing, recently recovered data [25]. As expected from the previous studies examining CVD biomarkers, the LA intervention group had lower levels of total cholesterol. However, unexpectedly this group had higher rates of death than controls (all cause 17.6% *vs.* 11.8%, hazard ratio 1.62 (95% confidence interval 1.00–2.64), *p* = 0.05; cardiovascular disease 17.2% *vs.* 11.0%, 1.70 (1.03–2.80), *p* = 0.04; coronary heart disease 16.3% *vs.* 10.1%, 1.74 (1.04–2.92), *p* = 0.04) once again raising the question of whether *n*-6 PUFAs may in some cases increase coronary heart disease.

It also has been recognized for more than a half a century that LA can be converted in humans to arachidonic acid (ARA) and this ARA can then be metabolized via cyclooxygenase and lipoxygenase pathways into eicosanoids such as prostaglandins, thromboxanes and leukotrienes. In general, eicosanoids have been shown to act as local hormones to promote acute and chronic inflammation in numerous human diseases including cardiovascular disease (encompassing atherosclerosis leading to heart disease and stroke) asthma, and arthritis [26]. Eicosanoids also have potent impact on bronchoconstriction, vascular permeability, platelet aggregation, and leukocyte recruitment, all postulated to contribute to several human diseases [27]. More recently, epidemiological, clinical and animal studies have provided evidence that ARA metabolism to eicosanoids is an important mechanism by which dietary fats impact carcinogenesis. Additionally, blocking the cyclooxygenase pathway mediating ARA metabolism with non-steroidal, anti-inflammatory drugs (NSAID) have been reported to have beneficial effects in reducing the risk of developing breast, colon, lung, and prostate cancer [28].

However, some metabolites of ARA are clearly not pro-inflammatory including the epoxyeicosanoids, while epoxyeicosanoid metabolites are deleterious. The pleiotropic properties of arachidonate metabolites as well as the inflammation resolving properties of certain metabolites of EPA and DHA make it difficult to completely understand the role of PUFA metabolites in inflammation [29,30,31]. Clarifying these concepts requires knowing which metabolites are made at local tissue sites at concentrations that regulate the cellular response we term inflammation.

Another critical, but much less discussed factor, in this debate is the potential interaction between ancestral genetic background and dietary PUFA environments, especially with regard to ARA synthesis and eicosanoid production. The scientific inquiry to date almost exclusively has examined the impact of dietary PUFAs found in the MWD in European or European American populations. However, recent studies discussed in detail below suggest that certain racial and ethnic groups (and especially those of African ancestry) have much higher frequencies of genetic variants in key genes that enhance their capacity to synthesize ARA and potentially eicosanoids. Many of these are the same variants that have been associated with CVD biomarkers and disease endpoints. Consequently, diet-gene interactions are likely to have a significant impact with regard to whether dramatically increasing dietary *n*-6 C18 PUFA, such as LA abundant in the MWD, is beneficial, slightly detrimental or a major risk factor for a given racial or ethnic group. Innis has [32] recently proposed that similar ancestral considerations must be taken into account with PUFAs in human breast milk and points out that that several recent studies suggest interactions between breastfeeding and genotype, child cognitive development, and risk of allergic disease.

### 2.3. Biosynthesis of LCPUFAs

As mentioned above, the primary *n*-6 LCPUFA, ARA can be synthesized from LA utilizing three (two desaturation and one elongation) enzymatic steps (Figure 1) [33]. The *n*-3 LCPUFAs, eicosapentaenoic acid (EPA) and docosahexaenoic acid (DHA) can also be synthesized from dietary ALA through seven (three desaturation, three elongation and one β-oxidation) enzymatic steps. The first three enzymatic steps that form ARA and EPA from LA and ALA, respectively, are thought to be carried out by the same three enzymes. EPA is then further converted to DHA utilizing additional biosynthetic steps (two elongation, one desaturation and one β-oxidation). Recent studies suggest that the efficiency of each of these steps in individuals is highly impacted by variants in the genes that encode for these enzymatic steps [34].

In addition to these biosynthetic pathways, LCPUFAs can also be obtained directly from the diet. Dietary ARA is sourced primarily from organ meats, eggs, poultry, and certain fish, whereas dietary EPA and DHA are found primarily in seafood [35]. Daily dietary intakes of ARA can range from as low as 50 mg to greater than 500 mg per day [35,36]. Consuming a meal of oily fish, such as salmon, albacore tuna, or mackerel provides roughly 500 mg to 2 g of *n*-3 LCPUFAs [37]. However, average *n*-3 LCPUFA consumption does not exceed 100 mg per day in the MWD. It is also well recognized that providing EPA and DHA as fish oil supplements enhances circulating and tissue levels of the *n*-3 LCPUFAs [38,39,40].

Given the shared enzymatic steps involved in the processing of LA and ALA, it is generally accepted that these *n*-6 and *n*-3 PUFAs and their metabolic intermediates compete with each other in the liver and other tissues as substrates for synthesis enzymatic reactions [41,42]. Specifically, LA and ALA are converted to gamma (γ)-linolenic acid (GLA, 18:3, *n*-6) and stearidonic acid (SDA, 18:4, *n*-3), respectively, by the enzyme encoded for by the gene fatty acid desaturase 2 (*FADS2*; chromosome 11q12.2). This has long been thought to be a rate-limiting step in LCPUFA biosynthesis [41,43,44]. Subsequently, GLA is elongated to dihomo-γ-linolenic acid (DGLA, 20:3, *n*-6) and SDA to eicosatetraenoic acid (ETA, 20:4, *n*-3) by an elongase step thought to be carried out by an enzyme encoded for by the gene elongase 5 (*ELOVL5*; chromosome 6p21.2–p12.1). DGLA and ETA are then converted to ARA and EPA, respectively, by an enzyme encoded by the gene fatty acid desaturase 1 (*FADS1*; chromosome 11q12.2–q13.1). This desaturase has also been proposed to be rate limiting [44,45]. However, it may be that since both *FADS2* and *FADS1* are closely aligned on chromosome 11, the expression of both is concomitantly, transcriptionally regulated [46]. In fact, Reardon and colleagues have suggested that there is an important regulatory region for *FADS1* transcription in the intron 1 of the *FADS2* gene [47].

EPA is eventually converted to DHA utilizing two elongation steps (both carried out by an enzyme encoded by the fatty acid elongase 2 gene *(ELOVL2*; chromosome 6p24.2), a desaturation *FADS2* step, followed by a β-oxidation step (the enzyme(s) for which and gene(s) that encode them have not been confirmed). *ELOVL5* may also participate in the elongation of EPA as there is significant overlap in the substrate specificities of *ELOVL5* and *ELOVL2* (both can utilize C20 LCPUFAs). However, only *ELOVL2* can convert the C22 *n*-3 LCPUFA, docosapentaenoic acid (DPA, 22:5, *n*-3) to 24:5*n*-3, which is the ultimate precursor of DHA [48]. The β-oxidation step, known as the Sprecher’s Shunt, is essential for the retro-conversion of a newly-synthesized 24 carbon *n*-3 PUFA to DHA [41,44,49,50,51]. The desaturation and elongation steps are thought to occur in the endoplasmic reticulum, whereas the β-oxidation step is thought to occur in peroxisomes [41,49,50,51]. While both ARA and EPA can be metabolized to longer (22–24) carbon PUFAs, examination of circulating and tissue levels of these LCPUFAs suggests the *n*-3 pathway is much more efficient at synthesizing 22 carbon LCPUFAs, DPA and DHA than the *n*-6 pathway [52]. This contention is supported by a recent study that shows *ELOVL2* activity exhibits a ~four-fold increase in substrate specificity for the *n*-3 substrate, EPA than the *n*-6, ARA [53].

Since LA and ALA share the same enzymes in the early steps of LCPUFA biosynthesis, the dramatic increase of LA and resulting *n*-6 metabolic intermediates as a consequence of the MWD might lead to reductions in the synthesis of *n*-3 LCPUFAs, further shifting to a higher *n*-6/*n*-3 LCPUFA ratio. Several studies support such a mechanism for reducing *n*-3 LCPUFA levels [16,17,18,54,55]. This reduction is not due to a decreased ALA consumption (in fact, ALA consumption has increased modestly over that same period of time), but is postulated to be due to the competition between LA, ALA and their intermediates down the PUFA biosynthetic pathway (Figure 1). The competition effect has recently been modeled in rat studies by Gibson and colleagues who demonstrated diets containing ALA can result in a substantial accumulation of circulating DHA, but only when the level of dietary LA is low in the diet [55]. At LA concentrations above 2% food energy, DHA synthesis from ALA is markedly reduced. This study is in close agreement with Hibbeln and colleagues who suggest that human *n*-3 PUFA ingestion could be reduced 10-fold if the intake of *n*-6 fatty acids, in particular LA, were lowered to 2% of energy [16]. In addition to substrate competition, there also has been a reduction in the dietary consumption of *n*-3 LCPUFAs that could also impact *n*-3 LCPUFA levels.

It has also been argued that in the face of such high concentrations of LA, the PUFA biosynthesis pathway from LA to ARA is saturated, limiting the amounts of ARA that can be made. This concept has been used to suggest that high dietary LA levels do not impact circulating or tissue levels of ARA [56,57,58]. This important point is addressed below with regard to the capacities of different racial/ethnic groups to synthesize ARA.

### 2.4. LCPUFA Cellular Metabolism

**Figure 2:**
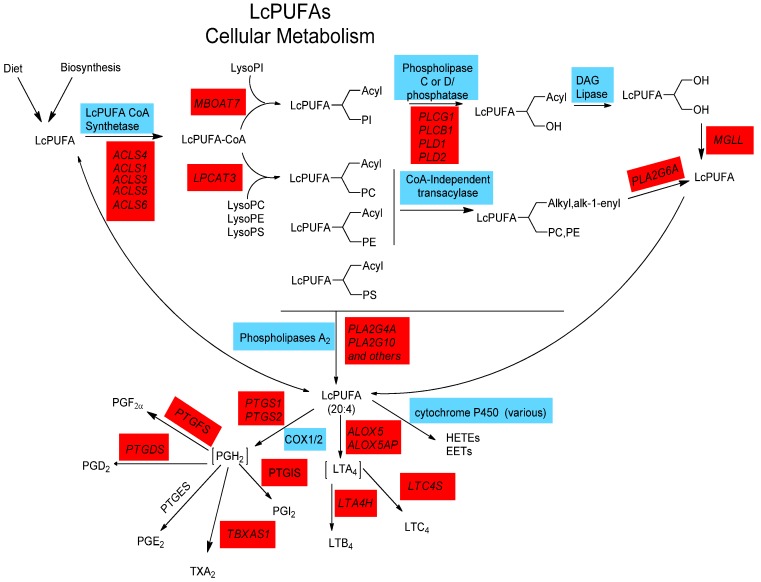
LCPUFA Cellular Metabolism. The incorporation of LCPUFAs into phospholipids and their release occur by a complex network of enzymatic activities and related genes. Free fatty acids are activated by conjugation with Coenzyme A (CoA) by gene products derived from up to five genes before incorporation into and remodelling through various phospholipids by enzymes differing in both LCPUFA and phospholipid acceptor specificities. Upon cell activation, phospholipases (A_2_, C and D) cleave esterified fatty acids from membrane phospholipids. Certain LCPUFAs (such as ARA) can then act as substrates for the eicosanoid-generating enzymes COX1/2, ALOX and P450.

Once LCPUFAs are synthesized or obtained from the diet, they are transported to cells and tissues in circulation as free fatty acids bound to albumin or esterified to complex lipids such as phospholipids, cholesterol esters, and triglycerides in lipoprotein particles [59]. A great deal remains unknown as to how LCPUFAs move through cellular membranes into the cell. However, once inside, they are acted upon by specific LCPUFA CoA synthetase(s) that converts free LCPUFA into LCPUFA-CoA to be utilized by LCPUFA-CoA: l-acyl-2-lysophosphoglyceride acyltransferase(s) to yield 1-acyl-2-LCPUFA phospholipids (Figure 2) [60,61]. The *ACSL4* gene is of particular interest in regard to the former activity as its gene product prefers LCPUFAs, with its specific activity being five-fold higher for ARA than for 18:1, *n*-9 [62]. With regard to the latter acyltransferase step, *MBOAT7* encodes for an enzyme activity that selectively incorporates LCPUFAs into lyso-phosphatidylinositol. In contrast, *LPCAT3* encodes an activity with broad specificity for lyso phosphatidylcholine (PC), phosphatidylethanolamine (PE) and phosphatidylserine (PS), but high activity for LCPUFAs [63]. There are several other CoA-dependent and CoA-independent enzymatic activities that may be responsible for the acylation of individual molecular species of a phospholipid class [64]. Of particular interest is a CoA-independent transacylase activity which catalyzes the remodeling of LCPUFAs from diacyl- to 1-alkyl and 1-alk-enyl-linked phospholipids. Additionally, a calcium-independent phospholipase A_2_ (PLA_2_), encoded by *PLA2G6A*, is a key enzyme that has been proposed to participate in the remodeling of LCPUFAs through membrane phospholipids [61]. Due to the complexity of these pathways, these participating enzymes and their genes unfortunately are often ignored. However, they will be critical to understanding diet-gene interactions as they control the degree to which dietary or synthesized LCPUFAs are made bioavailable for the signaling processes described below.

Figure 2 points out there are a large number of other genes that participate in LCPUFA metabolism including those responsible for incorporating LCPUFAs into cellular glycerolipids and subsequently remodeling LCPUFAs between glycerolipids. While there is little evidence to date that variation in these genes impact eicosanoid biosynthesis, disease biomarkers or endpoints, it is important to understand the entire PUFA system. It may be that employing a system-wide approach to the pathway examining potential gene-gene interactions will provide greater insights into the role of the entire pathway in human disease.

After LCPUFAs are incorporated and remodeled into cellular phospholipids, they can then be liberated from membrane phospholipids (typically after immunologic activation) as free fatty acids by a family of PLA_2_, diacylglyceride and monoacylglyceride lipases [65,66]. Two PLA_2_ activities that likely play a role in inflammatory responses are encoded for by genes such as *PLA2G4A* and *PLA2G10* genes [67]. Once released, LCPUFAs, particularly ARA, can then act as potent cell signals or be converted to a large family of eicosanoid products (including prostaglandins, thromboxanes, leukotrienes and lipoxins) via cyclooxygenase, lipoxygenase and cytochrome-P450 pathways (Figure 2). In addition, radical-based oxidation of LCPUFAs, whether free or esterified to phospholipids, is known to generate complex mixtures of additional products by non-enzymatic mechanisms (not shown in Figure 2). Some of these products are known to exert profound effects on the inflammatory response of cells [68].

### 2.5. Biological Roles for LCPUFAs Such as ARA, EPA and DHA

As outline above, ARA and its metabolic products play important roles in orchestrating immunity and inflammation [38,69,70,71]. This can occur via several mechanisms including their ability to directly impact normal and pathophysiologic responses through the conversion of ARA to potent eicosanoid products (including prostaglandins, thromboxanes, leukotrienes and lipoxins) (discussed above in Section 3 “A Debate about the Health Impact of *n*-6 PUFAs”). Alternatively, ARA and its oxidized products can regulate transcription, and consequently a wide range of cellular activities via cellular and nuclear receptors (such NF-κB, PPAR and SREBP-1c [69,72,73,74,75,76,77,78,79], thereby modulating the expression of many genes that control immune responses (cytokines such as IL-1, IL6, IL12 and TNF-α; chemokines such as IL-8, MIP-1α and MCP1; adhesion molecules such as ICAM and E-selectin; and inducer effector enzymes such as iNOS and COX-2) [71]. Alvheim and colleagues recently revealed another putative mechanism by which ARA may impact obesity and inflammation [80]. Specifically, they showed in mice, that increasing dietary LA from 1% to 8% of food energy consumed, significantly increased ARA in tissues as well as food intake, plasma leptin, and adiposity. These changes were associated with a three-fold increase in liver endocannabinoids, 2-arachidonoylglycerol (2-AG) and anandamide (AEA), two other bioactive ARA-containing mediators (not shown in Figure 2).

DHA is the most abundant fatty acid in the brain and retina, constituting a remarkable 50% of the weight of the neuron’s plasma membrane. A large body of scientific literature indicates that LCPUFAs are not only structurally important, but they are essential for proper brain function and development. DHA plays a critical role in neurogenesis and neural plasticity. Thus, adequate dietary DHA levels are essential for visual, neural and cognitive development in the developing fetus and young infants and perhaps recovery from neurological deficits [81,82,83].

DHA also has been shown to have potent anti-inflammatory properties. For example, DHA itself is known to directly inhibit NF-κB activation [84,85]. Additionally, Serhan and colleagues have demonstrated that increasing cellular uptake of *n*-3 fatty acids causes an enhancement in the production of DHA (and EPA)-derived resolvins and protectins [86,87,88], which are proposed to dampen and resolve inflammatory responses. EPA also has important anti-inflammatory properties and effectively competes with ARA for enzymes that participate in eicosanoid biosynthesis [89,90]. Specifically, increases in the EPA/ARA ratio of phospholipids within tissues following fish oil ingestion, may alter prostaglandin metabolism. Smith and colleagues demonstrated that increasing phospholipid EPA/ARA ratios in cells dampens prostanoid signaling with the largest effects being on PGHS-1 pathways involving prostaglandin D, prostaglandin E, and prostaglandin F [91]. Dietary supplementation with EPA, found in fish oil, also inhibits leukotriene generation, likely through substrate competition with ARA for the action of cytosolic PLA_2_ [91,92]. EPA is also converted to LTB_5_ that has about 100-fold less potency as a chemoattractant compared to LTB_4_ [93]. Finally, a recent metabolomics study revealed that feeding animals fish oils containing *n*-3 long chain PUFAs leads to a marked increase in levels of *n*-3 derived endocannabinoids with potential anti-inflammatory effects in several tissues, once again emphasizing the importance of the balance between *n*-6 and *n*-3 LCPUFAs [94].

### 2.6. Genetic Variations within the FADS Gene Cluster: Implications for Cardiovascular Disease

**Figure 3:**
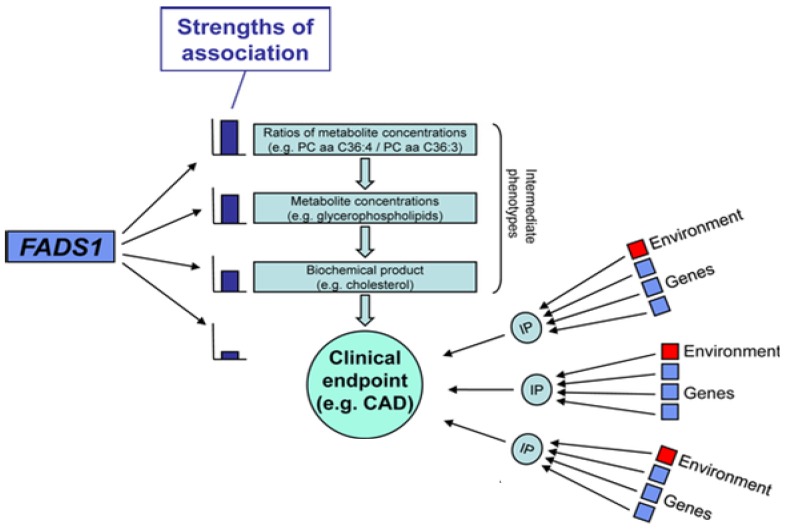
Associations between *FADS1* variants and intermediate, molecular phenotypes (IPs), such as metabolic traits. This figure illustrates that the strength of an association is related to the molecular step(s) and metabolite(s) most impacted by genetic variants. In this case, *FADS1* variants are most strongly associated with the conversion DGLA to ARA, and thus there are strong associations between the ratio of ARA-containing phospholipids to DGLA-containing phospholipids. This in turn impacts levels of circulating and cellular phospholipid, but the association with *FADS1* variants is not as strong because more molecular steps are involved. Levels of phospholipids in turn affects cholesterol levels in lipoprotein particles (as phospholipids and cholesterol esters within lipoprotein particles contain PUFAs) but again the associations are weaker due to the numerous factors (*FADS1* variants being only one) that effect total cholesterol levels. Adapted from Gieger *et al*. [98]

Three members of the fatty acid desaturase (*FADS*) gene family on chromosome 11q12–13 [95,96] include *FADS*1 and *FADS*2, demonstrated to code for the enzymes, Δ5 and Δ6 desaturase activities, respectively, while less is known about *FADS3*. Over the past decade, genome-wide association studies (GWAS) have identified a number of genetic polymorphisms that convey increased risk for coronary artery disease, diabetes, cancer, and other common diseases. Recent studies have combined GWAS analysis with the emerging area of metabolomics to more directly address intermediate molecular phenotypes (IP) involved in human diseases (Figure 3).

A central concept of these types of studies is that variation in DNA on its own does not lead to disease but, instead, alters molecular traits that go on to affect disease risk. By layering in molecular and intermediate phenotypes, causal relationships between genes and disease can be more directly established (reviewed in [97] (Figure 3)). Studies carried out by Geiger and colleagues showed that the combination of a GWAS (187,454 autosomal SNPs) and metabolomics (363 metabolites in serum) to be very powerful for identifying genes that participate in the synthesis of complex lipids that impact lipoprotein and cellular phospholipid metabolism [98]. As mention above (Figure 2), ARA-containing phospholipids are key components of circulating lipoproteins and cellular membranes. Geiger *et al*., showed that phospholipid metabolites with four double bonds (*i.e*., ARA) to be associated (most *p*-values ranging from 10^−3^ to 10^−8^) with SNPs in *FADS1* [98,99] and this effect was observed for all major phospholipid species (PC, PE, PI, including 1-acyl, 1-alkyl and 1-alk-1-enyl phospholipids) [98]. Moreover, the association with the SNP in the *FADS1* gene increases up to 14-fold (*p*-values below 10^−21^) when examining the ratios of putative PUFA-containing precursors and products. For example, the strongest effect size was observed with the ratio of ARA-containing PC to DGLA-containing PC (*p* = 2.4 × 10^−22^) with 28.6% of the total variance in the population being explained by one SNP, rs174548 in the *FADS* cluster. The authors point out that this effect is so strong that “if the molecular function of *FADS1* had not been already known, the association between the SNP and the different glycerophospholipid concentrations per se would have allowed (one) to deduce its enzymatic activity of inserting a fourth double bond” into DGLA to produce ARA.

In terms of more traditional markers of cardiovascular disease, two initial GWAS (focusing on SNPs in ~18,000 participants) demonstrated modest (1.89 × 10^−4^ and 6.07 × 10^−5^ *p* values) associations with the *FADS1* SNP, rs174548 and LDL-cholesterol, HDL-cholesterol, and total cholesterol levels [100,101]. More recently, Kathiresan and colleagues in a GWAS and a large replication study showed stronger associations between *FADS* cluster variants and both HDL-cholesterol (2 × 10^−12^) and triglycerides (2 × 10^−14^) [102]. Aulchenko also found strong genome wide significance (in 16 European population-based cohorts, *n* = 17,797–22,562) between variants in the *FADS* cluster and total cholesterol (*p* = 1.5 × 10^−10^) and LDL-cholesterol (*p* = 4.4 × 10^−13^) [103]. Teslovich and colleagues reported 95 common variants associated with plasma lipids in over 100,000 individuals of European ancestry [104]. The FADS variant, rs174546 was strongly associated with total-, HDL and LDL-cholesterol, and particularly triglycerides (5 × 10^−24^). However, there was no association with CVD. Perhaps, it is not surprising that there are such strong associations with *FADS* cluster SNPs and LC-PUFA-containing glycerolipids as well as total cholesterol, LDL-cholesterol, HDL-cholesterol and triglycerides given that PUFA-containing gylcerolipids are key molecular components (intermediate phenotypes) of lipoprotein particles (Figure 3).

Numerous studies have also examined the effects of SNPs in *FADS*1 and *FADS*2 on PUFA levels in both circulation and within cells in populations of European, Asian and more recently, African descent [105,106,107,108,109,110,111,112,113]. Schaeffer and colleagues first showed that common SNPs in the *FADS* gene cluster were associated with the PUFA composition of serum phospholipids in a European cohort [107]. They determined that minor alleles of SNPs in a region encompassing *FADS1* and the promoter of *FADS2* as well as the corresponding haplotype (comprising these minor alleles) were highly associated with an increase in levels of LA, eicosadienoic acid (C20:2*n*-6), DGLA, and ALA and a decrease in the levels of GLA, ARA, adrenic acid (C22:4*n*-6), EPA (*n*-3), and DPA (*n*-3). In a GWAS, Tanaka and colleagues demonstrated that the strongest genetic determinant of circulating plasma PUFA levels was a single SNP, rs174537 (*p* = 5.95 × 10^−46^) [114]. Minor TT allele homozygotes had lower ARA compared to the major GG homozygotes at rs174537, which accounted for 18.6% of the additive variance in ARA concentrations. While rs174537 maps to an open reading frame (*C11orf9*) 14.4 kb upstream of *FADS*1, this genomic region has extensive linkage disequilibrium and rs174537 maps to a single LD block, ~121 kb in size in European ancestry populations [112], including *FADS1*, *FADS2*, and most of *FADS3*. GWAS studies focusing on *n*-3 PUFAs in five population-based cohorts comprising 8866 subjects of European ancestry showed that variation in *FADS1* and *FADS2* to be associated with conversion of ALA to EPA. Interestingly, variation in the elongase gene, *ELOVL2* was associated with ratios of EPA or DPA to DHA, suggesting an important role for this gene in DHA biosynthesis [115]. We examined associations between genetic variants in the *FADS* cluster and LCPUFA synthesis in an island population with a homogenous dietary environment and found that variants pertaining to the *FADS1* step likely regulate the efficiency of conversion of C18 PUFAs to LCPUFAs, such as ARA. We observed a cluster of SNPs in tight linkage disequilibrium with the *FADS1* gene that were strongly associated with ARA, and the strongest associations were observed when examining *FADS1*-catalyzed conversion of DGLA to ARA. Taken together, there is strong, consistent evidence indicating SNPs within this region are strongly associated with plasma, red blood cell, tissue and breast milk LCPUFA levels. These data were recently reviewed by Lattka and colleagues [116].

There are also now numerous candidate gene studies which indicate that specific *FADS* haplotypes (favoring high desaturase activity) lead to enhanced levels of inflammatory and CVD biomarkers including oxidative products of ARA [105,117,118]. These then have been associated with CAD, myocardial infarction, and metabolic syndrome. In regard to CAD, Martinelli and colleagues first examined the association between 13 SNPs in the *FADS* cluster and PUFAs in plasma and erythrocyte membrane phospholipids in 876 subjects (610 cases and 266 controls) from the Verona Heart Study [119]. In addition to replicating previously-described *FADS* variant-PUFA associations, they demonstrated that a higher ARA/LA ratio (presumably representing more conversion of LA to ARA via the *FADS* cluster) is an independent risk factor for CAD (odds ratio: 2.55; 95% CI: 1.61, 4.05 for higher compared with lower ratio tertile; *p* < 0.001). Additionally, they linked ARA/LA ratios to systemic inflammation by showing that concentrations of high-sensitivity C-reactive protein (CRP) increased progressively across tertiles of ARA/LA. Importantly, increases in CRP concentrations and CAD risk were related to the *FADS* haplotypes associated with higher levels ARA and ARA/LA ratios.

Recently, Li and colleagues measured the association between plasma fatty acids and five *FADS* cluster SNPs in CAD patients (*n* = 505) and a control group (*n* = 510) of a Chinese Han population [120]. Once again, the ARA/LA ratio was higher in CAD patients and the low efficiency/capacity T allele at rs174537 was associated with a lower risk of CAD (OR 0.743, 95% CI (0.624, 0.884), *p* = 0.001). However, it is important to point out that not every population-based study has observed an increase in CVD with higher ARA levels. A recent prospective cohort study in the CAREMA, (a large Dutch cohort) demonstrated reduced CHD risk with increased ARA/DGLA ratios (*FADS1* activity) [121]. However, this same group showed that the reduced risk was also associated with increased DHA levels; DHA can be obtained in the diet as seafood or synthesized endogenously from ALA also utilizing *FADS1* activity. These contradictions may result from the interaction between the balance of C18 *n*-6 to *n*-3 PUFAs as well as the *n*-3 LCPUFA dietary environment and specific genotypes in the *FADS* cluster of the cohort being examined. For example, in a population that consumes more C18 *n*-3 PUFAs resulting in a lower ratio of LA to ALA, it may be that increased *FADS1* activity results in higher levels of protective *n*-3 LCPUFAs and this balance together with higher dietary DHA overrides increases in circulating and cellular levels of ARA. The key point here and throughout this review is that the impact of genetic variants are likely to be population-specific as both the frequency of the variants in PUFA biosynthetic genes and the dietary precursor PUFA environment are typically quite different from population to population. A precursor environment with relatively high concentrations of ALA relative to LA together with high efficiency/capacity variants may shift the balance of DHA, DPA, and EPA to ARA toward a wide array of protective *n*-3 containing eicosanoids and endocannabinoids (intermediate phenotypes). Alternatively, higher concentrations of dietary LA (relative to ALA) together with a high frequency of high efficiency/capacity variants likely shifts the balance back to ARA, proinflammatory eicosanoids and endocannabinoids, inflammation and associated human disease. The major point of this review is that given these complexities, simple recommendations are unlikely to be applicable to diverse populations.

ARA-derived eicosanoids, and particularly urinary 8-epi-prostaglandin F(2α) have been widely used as oxidative stress biomarkers and are widely recognized as a sensitive and independent risk factor for CAD [122,123]. Park and colleagues [124] have recently shown in a Japanese case-control study (control, *n* = 1123; CAD, *n* = 807) that CADs patients had higher levels of ARA and ARA/LA ratios in serum phospholipids. Additionally, they found a positive correlation between serum phospholipid ARA and urinary excretion of 8-epi-prostaglandin F(2α) in both controls and CAD patients [124]. Circulating ARA-containing phospholipids were also positively correlated with ox-LDL, a strong predictor for CAD. Similarly, Kwak and colleagues demonstrated in a Korean cohort that the minor T allele at the SNP rs174537 was associated with lower levels of ARA, higher levels of LA and a significantly lower number of CAD patients when compared to controls [118]. Importantly, that study also demonstrated that the proportion of ARA in serum phospholipids positively correlated with LDL-cholesterol, ox-LDL, and malondialdehyde in controls and with 8-epi-prostaglandin F(2α) in both control and CAD groups. A recent study by Hong and colleagues investigated associations between SNPs in the *FADS* cluster and age-related changes in PUFAs as well as oxidative stress and inflammatory markers in middle-aged non-obese men [125]. When monitored three years after an initial examination, the GG genotype at rs174537 was associated significantly increased ARA levels, LDL-cholesterol, ox-LDL, IL-6 and urinary 8-epi-prostaglandin F(2α) than the TT genotype. These data suggest that the GG genotype is not only associated with higher levels of these cardiovascular risk factors (intermediate phenotypes) at a selected point in time, but age-associated changes are also greater in the GG genotype.

An important limitation of the field as it currently exists is that little is known about how (the molecular mechanism by which) these SNPs or other genetic variation impact PUFA levels. While numerous studies use terms such as desaturase activities or efficiencies, these are not true activities (as would be measured with an isolated enzyme or subcellular fraction), but are typically ascertained by measuring PUFA precursor/product ratios within circulating or cellular lipids. Certainly, SNPs and other genetic variation in the *FADS* cluster could impact PUFA levels at a variety of levels including altering the sequence and enzymatic function of proteins arising from the *FADS* cluster and the expression of *FADS* cluster genes (via promoter usage, stability of transcript, *etc.*) [47,126,127].

While much of the discussion above has focused on the impact of genetic variation on LCPUFA biosynthesis, other mechanisms may play a key role in determining PUFA levels and biology. It is has long been recognized that dietary LCPUFAs impact the synthesis and metabolism of other LCPUFAs; for example, numerous studies show that *n*-3 supplementation reduces cellular AA levels [128]. Additionally, the conversion of ALA to DHA is known to be impacted by dietary concentrations of *n*-6 PUFAs [129]. Again, the molecular mechanisms behind these effects are largely unknown. The former has been hypothesized to be substrate competition (between ARA and DHA or EPA) for enzymes that participate in the acylation or remodeling of cellular glycerolipids and substrate competition and feedback inhibition by LCPUFAs of the biosynthetic pathway itself. There are likely other mechanisms (potentially epigenetic) that connect LCPUFA exposure to the transcriptional machinery of the PUFA biosynthetic pathway [126,129,130].

### 2.7. Interactions between PUFAs and Variants that Code for Enzymes that Mobilize and Metabolize Arachidonic Acid in Cardiovascular Disease

In addition to genes in the *FADS* cluster, there are numerous studies that support the concept that specific variants in genes whose activity are responsible for releasing ARA from membrane phospholipids (such as cytosolic PLA_2_ alpha; *PLA2G4*) or metabolizing ARA to eicosanoids (such as 5-lipoxygenase; *ALOX5*) may further be a source of gene-diet interactions and predispose individuals with high amounts of *n*-6 PUFAs to enhanced inflammation and cardiovascular disease [131,132,133]. Once ARA is released from glycerolipids via a PLA_2_ cleavage, 5-lipoxygenase (*ALOX5*) becomes the rate limiting step in leukotriene generation from ARA (Figure 2). Since the original identification of “slow reacting substance of anaphylaxis” as a class of leukotrienes, this group of eicosanoids has primarily been identified with inflammation and bronchoconstriction associated with asthma and allergies. Additionally, a large body of evidence has emerged over the past decade that demonstrates that leukotrienes play a key pro-athrogenic role particularly in CVD [134,135,136].

In 1999, Drazen and colleagues demonstrated a pharmacogenetic association between tandem Sp1 binding sites in 5-lipoxygenase (*ALOX5)* and the response of asthmatics to treatment [137]. Subsequently, Dwyer and colleagues examined carriers of common verses rare variant alleles (6% of the population) with regard to tandem Sp1 motifs in the *ALOX5* promoter in a cohort from Los Angeles [138]. Specifically, they demonstrated that individuals with variant genotypes in the *ALOX5* promoter had a significantly higher level of carotid atherosclerosis and risk of myocardial infarction. A potentially important diet-gene interaction was identified in that study when it was shown that dietary ARA intake significantly enhanced the atherogenic effect of these *ALOX5* genotypes. Another study [139] demonstrated in a cohort from Costa Rica that variant alleles with 3 or 4 sites (or, rarely, >5 sites) were associated with greater intima-media (IMT) thickness of the carotid artery and with occurrence of a first myocardial infarction. As with the former study, dietary ARA significantly enhanced IMT thickness and the occurrence of a first myocardial infarction [139].

As mentioned above (Figure 2), cellular ARA must be mobilized from glycerolipids via a PLA_2_ reaction before it can be converted to bioactive eicosanoids. While there are many isoforms of PLA_2_, most investigators in the field agree that Group IV PLA_2_ (*PLA2G4*) plays a critical role in ARA release from phospholipids. Similar to *ALOX5*, two recent studies by Hartiala and colleagues have observed that a genetic variant in *PLA2G4* is associated with a decreased risk of myocardial infarction in of Northern European and Costa Rican populations [140,141]. Further nutrigenomic analysis in the Costa Rican population demonstrated that the cardioprotection associated with the gene variant rs12746200 occurs primarily in AG/GG subjects who had high dietary LA intakes [141].

### 2.8. Potential Interactions between Dietary PUFAs and Genetic Variants in the FADS Cluster and other PUFA Metabolizing Genes; a Potential Contributor to Health Disparities

As mentioned in the Introduction, African Americans have higher rates of hypertension, type 2 diabetes, stroke, and CVD and certain types of cancer than Caucasians. Not only is the incidence of these diseases elevated among African Americans, but the diseases themselves are more severe in African Americans than in Caucasians [10,14,15]. The key question of this review is could enhanced ARA synthesis and metabolism in populations of African descent contribute to disparities observed in inflammatory disease? There are several lines of reasoning that support this hypothesis. First, ingestion of a MWD diet, highly enriched in LA, together with specific genetic determinants (e.g., *FADS* variants that favor higher desaturase activity) increase the synthesis and bioavailability of circulating and tissues levels of ARA, and this is observed to a greater degree in populations of African descent. Second, higher levels of ARA within circulating and cellular glycerolipids enhance the synthesis of elevated levels of ARA-derived pro-inflammatory eicosanoids and other CVD biomarkers; and third, the MWD combined with specific genetic variants in the *FADS* cluster observed more often African ancestry populations are associated with higher incidences of human disease.

With regard to the first, the studies described above calls into question one of the generally accepted tenants of the PUFA field, that the ARA synthesis pathway saturates itself (presumably at the desaturation steps) in all humans at relatively similar and relatively low levels (2%–3% energy) of dietary LA. Thus, it is presumed that higher levels of dietary LA (in the MWD) have no impact on circulating or tissue levels of ARA. A recent review by Johnson and Fritsche concluded that there was little evidence that addition of LA to the diet increases the concentration of inflammatory markers in healthy humans [142]. However, this review had several critical limitations, but most importantly, only one of the 15 studies may have utilized subjects other than European or European ancestry populations; even this one South African study did not reveal the racial composition of subjects. These studies had other important limitations including short study durations (2 weeks to 40 days), and the average sample size for the studies was 33. Similarly, at least five other studies have shown that increasing dietary LA intake above 3% of energy has no impact on circulating and tissue ARA levels [143,144,145,146,147]. However, these too were carried out in small numbers of volunteers from Europe (Netherlands, Spain, Germany), Canada (British Columbia) and Australia. Liou and Innis carried out a carefully-performed study in Canadian (British Columbia) volunteers and found that plasma levels of ARA are increased as dietary LA is increased from 0 to ~3.5% energy and that further increases no longer impact levels of plasma ARA [147]. Increasing dietary LA above 3.5% did reduce plasma levels of *n*-3 LCPUFAs such as EPA and also increased levels of the elongation product of LA, eicosadienoic acid (20:2). It was interesting to note that even within such a homogeneous population, there was considerable variability in plasma ARA levels that could not be explained. However, in fairness to these investigators, this study was carried out before the impact of variants in the *FADS* cluster was widely appreciated and even then, the authors stated that “tight control of ARA among individuals in our studies may reflect genetic rather than dietary variables” [147]. These estimates of LA saturation of the desaturation/elongation pathway at ~3.0% energy are in agreement with those proposed by Lands [148].

Unfortunately, there have not been studies investigating the impact of dietary LA on ARA levels and whether this is related to disease biomarkers (including eicosanoids) and endpoints of African ancestry populations. To more fully address the issue of dietary LA and its capacity to saturate the desaturation/elongation pathway, similar metabolic studies to those described above are desperately needed in other (than European ancestry) populations. Such studies would shed light on whether higher proportions (than 3% energy) of dietary LA are converted to ARA in populations that have high frequencies of genetic variants in the *FADS* cluster associated with higher ARA levels.

Our studies have focused on comparisons between African and European American populations and uncovered dramatic differences in both circulating levels of ARA and frequencies of key SNPs within the *FADS* cluster [112,113,149]. Specifically, we have found higher levels of ARA and ratios of ARA to precursors (DGLA or LA) in African-American individuals compared to European-Americans. These differences are highly associated with genetic variation in the *FADS* gene cluster. Specifically, these studies show that ~85% of African Americans contain the GG genotype at rs174537 while ~43% of European Americans contain this genotype. This is the SNP in the *FADS* cluster that has been most highly associated with ARA levels (*p* = 5.95 × 10^−46^) (113). In our studies, African American individuals with the GG genotype have an average of ~10% of total circulating fatty acids (includes ARA-containing free fatty acids, phospholipids, triglycerides and cholesterol esters) as ARA, and European Americans with the GG genotype have ~8% of total circulating fatty acids as ARA. Non-GG individuals have circulating ARA levels ranging from 5% to 8% of total fatty acids, again with African Americans having higher levels [112,113]. As pointed out above, while there were identical estimated allelic effects of rs174537 on ARA levels in African Americans and European Americans, there was also a consistent difference in the predicted ARA mean within each genotype group between the races [112]. This observation of higher ARA values between African Americans and European Americans within each genotype suggests in addition to *FADS* genotype, there are likely other factors that drive ARA levels higher in African Americans. Taken together, these data raise doubts as to whether the conversion of dietary LA to circulating ARA is saturated at relatively low concentrations of dietary LA (~3% as proposed above) in the majority of individuals in all racial/ethnic groups and suggest that genetics may play a key role. If the conversion of dietary LA to ARA is saturated at higher levels (above 3% of energy) in African and European ancestry individuals with the GG genotype and additional factors further drive this conversion in African ancestry populations, then recommendations of dietary LA at 5%–10% of energy may not be appropriate for these populations.

With regard to the other questions of whether the *FADS* variants lead to an increase in intermediate phenotypes of CVD, including circulating ARA-containing phospholipids, lipoproteins (total cholesterol, LDL-cholesterol, HDL-cholesterol and triglycerides), inflammatory biomarkers (CRP and eicosanoids) as well as a higher incidences of cardiovascular disease in African Americans, the answer has not been directly addressed due to the lack of studies in these populations. However, the data are consistent that European or European-American individuals having those *FADS* cluster variants observed at much higher frequencies in African ancestry populations, are at significantly higher risk of having higher levels of the aforementioned intermediate molecular phenotypes as well as CVD [97,98,99,100,101,102,103,104,105,106,117,118,119].

There is also the troubling potential for the combination of variants in more than one gene within the PUFA biosynthetic and metabolic pathways (Figure 1 and Figure 2) to markedly increase intermediate phenotypes and disease incidence. For example, metabolomics studies make it clear that certain *FADS* variants (those found at higher frequency in African ancestry populations) are highly associated with ARA-containing phospholipids levels including 1-acyl, 1-alkyl and 1-alk-1-enyl linked phospholipids [98]. This reveals that the impact of the variant is observed through the ARA-CoA synthetase step as well as the CoA-dependent and independent acylation steps to enhance the synthesis of bioavailable ARA (within circulating and cellular phospholipids). With regard to the eicosanoid production, these phospholipids are now loaded with elevated levels of ARA waiting for cell activation and PLA_2_-dependent ARA cleavage.

We do not yet know if there are subsequent enzymatic steps in the cascade for which gene variants, in *PLA2G4*, *ALOX5* or *PTGS2* for example, may impact biosynthetic efficiencies and result in varied lipid mediator levels among different populations. In fact, to our knowledge, there have been no studies which have compared the capacity of different populations to generate eicosanoids (from lipoxygenase and cyclooxygenase pathways, or non-enzymatic mechanisms). However, a recent study suggested that this potentially harmful scenario is likely [140]. This study examined the shorter “3” and “4” Sp1 repeats of alleles in the *ALOX5* promoter region in both Caucasian and African American subjects. The shorter “3” and “4” repeats of alleles in the *ALOX5* promoter region increased CAD in only African Americans [140]. A likely explanation for the adverse effect on CAD is that African Americans exhibit a 10-fold higher frequency of the “3” allele compared to Caucasians (~30% compared to 1%–2%). As mentioned above, polymorphisms in *ALOX5* can be highly influenced by the dietary ARA levels [138]. The fact that these variants (like the high efficiency/capacity *FADS* variants) are much more prevalent in African Americans potentially set up a two-step mechanism that predisposes African Americans to CAD. The first step is provided by an increased frequency of genetic variants in the *FADS* cluster that leads to bioavailable ARA. This is then followed by an enhanced likelihood of elevated leukotriene levels as a result of an increased frequency of high leukotriene producing alleles.

Based on the previous observations, this same group evaluated the effect of *ALOX5* repeat genotypes on fish oil supplementation in people of African ancestry [150]. As predicted, supplementation led to an increase in EPA, DHA and total *n*-3 LC-PUFA in red blood cell membranes in most subjects. However, individuals with the high (cardiovascular disease) risk “dd” genotype did not respond to supplementation with an increase in RBC *n*-3 LC-PUFAs. This surprising observation suggests that not only are there interactions between these gene variants and the *n*-6 PUFA levels, but that these at-risk repeat gene variants are somehow interacting with *n-*3 PUFAs.

### 2.9. How Did These Ancestral Differences Arise?

Ameur and colleagues [151] as well as ourselves [149] have recently demonstrated large geographic variance, with a high frequency of a haplotype with an increased capacity to synthesize LCPUFAs in African and African ancestry populations and a loss of that converting capacity across Asia into North and South American native populations. The former paper identified two haplotypes (A and D) based on desaturase activity efficiency and showed that the high efficiency haplotype to be fixed in Africa, while Europe was ~75%, Eastern Asia and Oceanic region ~50% and <5% in native Americas populations.

We examined genetic variants in 1092 individuals from 14 different populations. Similar to Ameur *et al*. [151], we found dramatic differences in the frequencies of variants in the FADS cluster between African verses non-African populations [149]. These data revealed recent positive selection along a 1Mb region on chromosome 11q12–13 in the window containing the SNP rs174537 within Africa, with no evidence for selection in either Europe or the Americas. Simulations suggested that the target locus is likely to be within 50 kb of the signal and that a selective sweep at or near rs174537 within the African continent was likely complete or nearly complete. Median-joining network visualization of the haplotypes within this block suggests that the selection process occurred ~85,000 years ago [149].

**Figure 4:**
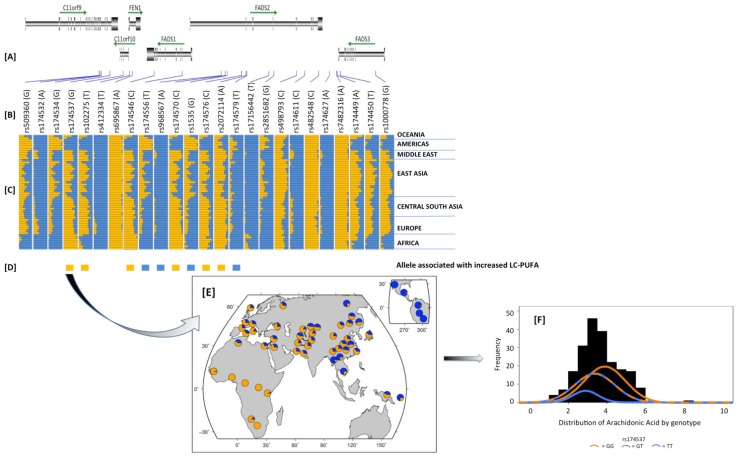
Dramatic differences in the frequency of derived alleles in a 100 kb region surrounding rs174537 in the Human Genome Diversity Panel Data. (**A**) is the physical location of the SNPs and genes in the region; (**B**) is SNP name and the specific derived allele in parenthesis; (**C**) is the derived allele frequency in orange in 52 populations clustered based on geography; (**D**) reflects the allele associated with increased LC-PUFA metabolism from published studies.; (**E**) is the detailed overview of rs174537; (**F**) shows three distributions within the Tangier Island Population. Adapted from Mathias *et al.* [149] (Panels **A**–**E**) and from Mathias *et al* [111]*.* (Panel **F**).

This work confirmed marked global differences in the allele frequencies of variants in the *FADS* gene cluster first noted in our work on African Americans and European Americans, especially at variants strongly associated with the efficiency of conversion of PUFAs. Figure 4 is a detailed synopsis of the patterns of genetic variation in a 100 kb region within the *FADS* gene cluster and illustrates three points: (1) clearly there are wide differences in the frequencies across numerous SNPs between populations, this is a function of extensive linkage disequilibrium in this region; (2) for SNPs for which allelic effects on LC-PUFA metabolism are known, the specific alleles that favor enhanced LC-PUFA metabolism across populations typically increase with an increasing African admixture component; and (3) rs174537 is notable in that the derived allele is the allele fixed within Africa. It is not clear why the positive selective pressure on the high efficiency *FADS* variants were lost after the expansion of populations from Africa. We have speculated that once LCPUFAs could be obtained in the diet due to the emergence of hunting, fishing, animal husbandry and other technological advances, the pathway may have lost its selective advantage. In any event, the world is now left with large diverse populations in countries such as the US, which are particularly distinct with regard to the frequency of important *FADS* cluster variants and potentially their capacity to synthesize *n*-6 LCPUFAs from very high levels of *n*-6 C18 PUFAs and particularly LA found in the MWD.

## 3. Conclusions

The body of work cited in this review indicates that the impact of increased dietary LA (observed over the past 50 years) on CVD and other human diseases is likely to be extremely complex. Major differences in the frequencies of variants that enhance the synthesis and metabolism of ARA within Americans of different ethnic descents make the possibility of uniform nutrition recommendation unlikely. Understudied populations (such as those of African descent) create a vital challenge with regard to formulating proper recommendations of dietary *n*-6 PUFAs. In addition to CVD, there are other diseases including several cancers such as prostate and colon cancers where animal models have provided strong evidence that *n*-6 PUFAs drive the initiation and progression of these cancers. Moreover, blocking ARA metabolism with NSAIDs reduces the risk of humans developing these cancers. Importantly, many of these same cancers disproportionately impact Americans of African descent [12]. Unfortunately, none of the presented or available literature has evaluated the importance of gene-PUFA interactions in diverse populations; this may ultimately be the most critical missing piece to the review in its present form. Clearly, studies designed to examine either the role of genetics in a controlled dietary environment or ones that evaluate these interactions by accounting for current dietary exposures are necessary in the future. Of immediate interest in lieu of the aforementioned complexities is the 2009 American Heart Association recommendation for humans to consume at least 5%–10% of energy intake by way of *n*-6 PUFAs. Given the potential risks, we feel that further research is needed before dietary recommendations for dietary LA should be made for heterogeneous populations that make up this and many other nations. It will be decades before we understand the impact of the MWD on human health and disease. However, we can say with some certainty that given the marked differences in ancient adaptive responses based on a population’s distinct ancestry and early diet, the MWD will likely effect certain segments of modern populations more negatively than others.

## Abbreviations

ALA: α-linolenic acid
ARA: arachidonic acid
COX: cyclooxygenase
DGLA: dihomo γ-linolenic acid
DHA: docosahexaenoic acid
EPA: eicosapentaenoic acid
ETA: eicosatetraenoic acid
CAD: coronary artery disease
CVD: cardiovascular disease
FADS: fatty acid desaturase
GLA: γ-linolenic acid
GWAS: genome wide association study
IMT: intima-media thickness
LA: linoleic acid
LC: long chain
LD: linkage disequilibrium
LTB4: leukotriene B4
C18: 18 carbon
MWD: modern western diet
NSAID: non-steroidal anti-inflammatory drug
PC: phosphatidylcholine
PE: phosphatidylethanolamine
PI: phosphatidylinositol
PS: phosphatidylserine
PLA_2_: phospholipase A_2_
PUFA: polyunsaturated fatty acid
SDA: stearidonic acid
